# Random Network Models to Predict the Long-Term Impact of HPV Vaccination on Genital Warts

**DOI:** 10.3390/v9100300

**Published:** 2017-10-16

**Authors:** Javier Díez-Domingo, Víctor Sánchez-Alonso, Rafael-J. Villanueva, Luis Acedo, José-Antonio Moraño, Javier Villanueva-Oller

**Affiliations:** 1 FISABIO-Public Health, 46020 Valencia, Spain; diez_jav@gva.es; 2 Instituto Universitario de Matemática Multidisciplinar, 8G building, 2nd Floor, Camino de Vera, Universitat Politècnica de Valéncia, 46022 Valencia, Spain; vicsana3@doctor.upv.es (V.S.-A.); rjvillan@imm.upv.es (R.-J.V.); jomofer@imm.upv.es (J.-A.M.); 3 Departamento de Ciencias de la Computación, Arquitectura de Computadores, Lenguajes y Sistemas Informáticos, Estadística e Investigación Operativa, Universidad Rey Juan Carlos, Móstoles, 28933 Madrid, Spain; javier.villanueva@urjc.es

**Keywords:** human papillomavirus, network model, vaccination strategies

## Abstract

The Human papillomaviruses (HPV) vaccine induces a herd immunity effect in genital warts when a large number of the population is vaccinated. This aspect should be taken into account when devising new vaccine strategies, like vaccination at older ages or male vaccination. Therefore, it is important to develop mathematical models with good predictive capacities. We devised a sexual contact network that was calibrated to simulate the Spanish epidemiology of different HPV genotypes. Through this model, we simulated the scenario that occurred in Australia in 2007, where 12–13 year-old girls were vaccinated with a three-dose schedule of a vaccine containing genotypes 6 and 11, which protect against genital warts, and also a catch-up program in women up to 26 years of age. Vaccine coverage were 73% in girls with three doses and with coverage rates decreasing with age until 52% for 20–26 year-olds. A fast 59% reduction in the genital warts diagnoses occurred in the model in the first years after the start of the program, similar to what was described in the literature.

## 1. Introduction

Human papillomaviruses (HPV) encompass more than 100 genotypes of viruses that infect cutaneous, genital and respiratory epithelia of humans worldwide. These are divided into high risk (HR), those that are directly related to the origin of cancers, and low risk (LR) genotypes that provoke mucocutaneous lesions. HPV 16 and 18 are responsible for about 70% of cervical cancers, and different proportion of other malignancies: vaginal, anal, mouth and throat, etc. HPV 6 and 11 are responsible for over 90% of genital warts (GW). High risk genotypes are the direct cause of clinically important diseases such as cervical carcinoma associated with oncoviruses HPV 16 and 18. In recent years, about 500,000 new cases were diagnosed annually and 200,000 women died as a consequence of the progression of cervical dysplasias to invasive carcinomas [1].

HPV is the most common sexually transmitted infection (STI) in the world. It is estimated that about 1 million new cases of GW are reported each year and the cost of treatment is increased by the tendency of these warts to recur after initial clearance. The cost of the treatment of genital warts was estimated to exceed $ 3.8 billion in the U.S. in 1997 [2]. In Spain, there were 35,000 cases in women in 2007 with an overall annual cost of € 47 million [3].

Two vaccines consisting of non-infectious HPV virus-like particles (VLP) [4] containing the capsid protein L1 of the virus but not the viral DNA. They induce a high immune response and prevent premalignant lesions. Both vaccines contain genotypes 16 and 18, and one of them adds VLP against genotypes 6 and 11 (HPV4).

In the Region of Valencia, Spain, the vaccine is administered to girls aged 15 years old or less. Similar vaccination strategies of this kind were modelled by Elbasha et al. [5,6] by means of a compartmental model with 17 age groups for each gender. This model focuses mainly on the development of cervical intraepithelial neoplasia (CIN) and its progression from CIN1 to CIN3. According to these authors, vaccination must be implemented for adolescent girls aged between 12 and 14 years. Elbasha et al. also found some evidence that the vaccination of boys could also be cost-effective [5]. By vaccinating girls alone, an 83% reduction in the incidence of GW is expected, but this reduction is increased to 97% if boys are also vaccinated.

In recent studies [7,8], a decrease in the number of infected persons and the number of persons with GW is already reported in Australia after two years of administering vaccinations to young girls. It showed both direct and indirect protection in males. These results were more impressive than the predictions of the continuous models. A Bayesian model for HPV vaccination was then proposed by Bogaards et al. [9] and focused on the herd immunity effect of the female vaccination on the male population in a static picture. A dynamic understanding for the short- and long-term effects of vaccination policies is, however, still necessary and even more so with HPV vaccines because their benefit to the whole population is to be observed in the time span of several decades.

New vaccination schedules, especially vaccination in boys, should take into account the herd immunity effect vaccination in girls (in mainly heterosexual societies); therefore, models that simulate STI would be important. Network models are those models in which the relations of the individuals (represented by network sites) are taken into account by means of network bonds. Random network models have been successfully applied to other infectious diseases such as the Respiratory Syncytial Virus pandemic [10] as well as other social pandemics such as obesity [11].

STI are more likely to produce large-scale infections than other transmissible diseases, such as respiratory transmitted diseases, because the efficacy of sexual contacts for the infection is large and the infectious agent has long latency periods as in the case of HIV or HPV. Moreover, neither the carrier nor his/her partner are aware of their exposure. For example, it has been estimated that around 40–50% of contacts are capable of transmitting HPV [12]. On the other hand, some STIs are caused by oncoviruses such as Hepatitis B or HPV, which increase the death rate of people who develop the disease.

In order to predict the evolution of these diseases, we need a reliable model of the underlying social network in which the infection builds up. Individuals who change partners or have several partners simultaneously are the hubs favouring the spread of STI. The distribution of degrees of the nodes in the network and the average chemical path from an infected individual to a susceptible one, are important parameters controlling the final extension of a new STI in a population and the speed at which it spreads. However, most models are based on some assumptions, which could not be valid for certain populations. Some studies claimed that the web of human sexual contacts is a scale-free network characterized by a power-law distribution for the number of individuals with a certain degree of connectivity, *k*: $P\left(k\right)\propto 1/k^{\alpha }$ with a value of α in the range 2<α<3, and slightly smaller for males than for females [13]. Although P(k) provides some valuable information about the network, it is not a sufficient prescription on how to build it for a given population size. Moreover, a power-law distribution of contacts could not be representative of some populations, or could vary from country to country.

Some field studies have ascertained the structure of moderate size real networks of sexual contacts. In 2004, Bearman et al. published the results for a set of 800 adolescents in a midsized town of the United States [14]. They showed that the structure of this network is a big cluster with a ring and extended filaments, which contained most of the adolescents implying that, potentially, the infection of an individual could spread to the whole of the population, given sufficient time and infectivity. A similar study was performed in 2007 at the Likoma Island in Malawi with the idea of predicting and explaining the expansion of HIV in sub-Saharan populations [15]. This study disclosed that the sexual network contained many cycles, in contrast with the single cycle at Jefferson High School. For this reason, it was speculated that superimposed cycles could be the explanation of the high prevalence of HIV infection in small populations of Africa.

Some recent studies reveal that the evolution of partnerships is also an important factor in the transmission of STIs. In particular, they pointed out that the following items should be considered: (i) the cumulative distribution of the lifetime number of partners, (ii) the distribution of partnership durations, (iii) the distribution of gap lengths between partnerships, and (iv) the number of recent partners. A method for building up networks considering these items has been developed by Schmid and Kretzschmar [16]. However, this information is not available in most surveys, and we therefore face the problem of developing reasonable models for STIs in many countries where information about sexual behaviour is scarce. For example, in the case of Spain, there is only available data about the number of sexual contacts in a lifetime from surveys. This is sufficient for building a sexual network for the transmission of HPV or other diseases with lifetime consequences and progression. In these cases, the important fact is whether the individual has had a contact with risk of infection. The remaining aspects of the network such as the duration of partnership and the time intervals among them can be incorporated effectively into a probability of transmission parameter.

In this paper, we show how to build a network model for sexual contacts from the usual statistical data in surveys concerning the number of partners in a lifetime. We consider both heterosexual men and men who have sex with men (MSM) populations and the connections among them. We perform simulations over this network substrate on the HPV infections by different genogroups including both LR and HR infections. In particular, we show that for the case of Australia the strategy of a vaccination for 12–13 year-old girls plus catch-up lead to a considerable reduction in the number of cases of infection by HPV 6 and/or 11 (which are the main cause of GW). For women in the 14–26 age-group, we obtain a decrease of 59% after 3.6–4.6 years and 39% in men after 3–3.75 years. These results agree with the conclusions of the study by Ali et al. [8].

## 2. Materials and Methods

### 2.1. Origin of the Data

Demographic data from the region of Valencia (Spain) was collected from the Valencian Institute of Statistics (2013) [17]. Lifetime sexual partners for an individual (LSP) was obtained from the Health and Sexual Habits Survey of 2003 [18], and summarized in Table 1.

Some features of the distribution of contacts were: (i) the percentage of males and females with no partners is very similar in each age-group; (ii) the proportion of women with a single partner is, approximately, two times larger than men with only one partner; and (iii) the percentage of men with two or more partners is always larger than that of women except for women in the age-groups 14–29, and 30–39 in the case of two partners. The asymmetry in the behaviour of males and females should be taken into account in the construction of the network.

### 2.2. Network Model

Before proceeding with the definition of our model, it is convenient to give a brief and general perspective on the emergent field of network research for the readers not familiar with these techniques and their application in epidemiology. A network is, basically, a model which derives from the abstract mathematical concept of a graph composed by a set of points (the so-called nodes) connected among them by some lines or edges (known as “links” in the case of networks). There are several types of networks of interest for the applied sciences. If we classify them according to the degree of a node, i.e., to the number of links for a given node (or, more properly, to the distribution of these number of links), we have two main categories in the literature: (i) random networks, in which the links or edges occur with a fixed probability and the statistical distribution of this number of links follows a Poisson’s law; and (ii) scale-free networks whose distribution of degrees follows a power-law with an algebraic tail of the form $P\left(k\right)≃1/k^{\gamma }$ with 2<γ<3. This means that the nodes with very large degrees are more likely to appear in scale-free networks than those in random networks. Random networks have been used in epidemiology [10] and also as an elemetary model of the brain [19]. On the other hand, scale-free networks have been successfully applied to the Internet and biological networks in which some nodes with a very large number of links are determinant in the control of the dynamics (see [20]). Small-world networks are also an important paradigm in the science of networks. This concept refers to the average length of a path connecting two typical nodes in the network. It was found that some sparse networks may, on the other hand, present the small-world phenomenon, i.e., we have short paths connecting every pair of nodes through the links with other nodes. A mechanism to generate these networks was discovered by Watts and Strogatz [21]. Many networks in sociology exhibit this small-world property [11,13,14].

In this paper, we use the random network model as a basis to simulate the network of sexual contacts among individuals, but, in this model, the average number of connections depend upon the age-group as deduced from Table 1. A basic property of the network we are going to discuss is that the total number of lifetime sexual partners (LSP) for the male population (M) must coincide with the total number of LSP for the female population (F). This is so because (in a purely heterosexual network) every link starting on a male must end in a female and viceversa. In mathematical terms:

(1) $$\sum_{i=1}^{M}\;LSP_{i}=\sum_{j=1}^{F}\;LSP_{j}\;.$$

The estimation of sexual partners: there are some approaches to the number of LSP in males and females [22,23] that are difficult to match. Generally speaking, males tend to overestimate the number of their sexual partners and females tend to underestimate it. Therefore, we considered the average LSP male value, ${\langle k\rangle }_{m}$, and calibrated the network so that results were consistent with data of Table 1, and estimated that the number of LSP in males in Spain was at least 4.5. Networks with 250,000, 500,000 and 750,000 have been necessary to perform the present study. It required a substantial computational power.

### 2.3. Semi-Random Construction

From Table 1 (proportion of male LSP aged 14–29), we have the following list: (0.107,0.314,0.445,0.67,0.838,1) for the accumulated proportion of males less than or equal to a given LSP number. Now, we randomly generate a number *r* between 0 and 1 and assign the number of contacts to every male node in the 14−29 age group, in the network as follows:

- r≤0.107 say that the corresponding male does not have an LSP,
- 0.107<r≤0.314 say that the corresponding male has one LSP,
- 0.314<r≤0.445 say that the corresponding male has two LSPs,
- 0.445<r≤0.67 say that the corresponding male has three or four LSPs uniformly distributed,
- 0.67<r≤<0.838 say that the corresponding male has five to nine LSPs uniformly distributed,
- 0.838<r≤1 say that the corresponding male has 10 or more LSPs.

Every node in the network is labelled by its gender and age randomly assigned according to the population histogram. The assignment of the number of bonds, as another label of the node, is not so straightforward since we must guarantee that the condition in Equation (1) is verified. In order to fulfill this condition, we take advantage of the uncertainty of statistics reports concerning individuals with 10 or more LSPs. Starting with the males, we assign the number of LSPs up to nine partners and, for 10 or more partners proceed as follows: let $i_{\max}$ be the number of males with nine or less partners. The unassigned males should be $M-i_{\max}$ and the number of bonds that should be distributed among them is $M{\langle k\rangle }_{m}-\sum_{i=1}^{i_{\max}}\;LSP_{i}$. By Euclidian division, this quantity can be expressed as $\left(M-i_{\max}\right)n_{m}+r_{m}$, where $n_{m}\geq 10$. In our procedure, we assign a random number of bonds, uniformly distributed, in the interval $\left[10,2n_{m}-10\right]$ to every male with 10 or more LSPs, i.e., to the $M-i_{\max}$ unassigned males.

**Figure 1:**
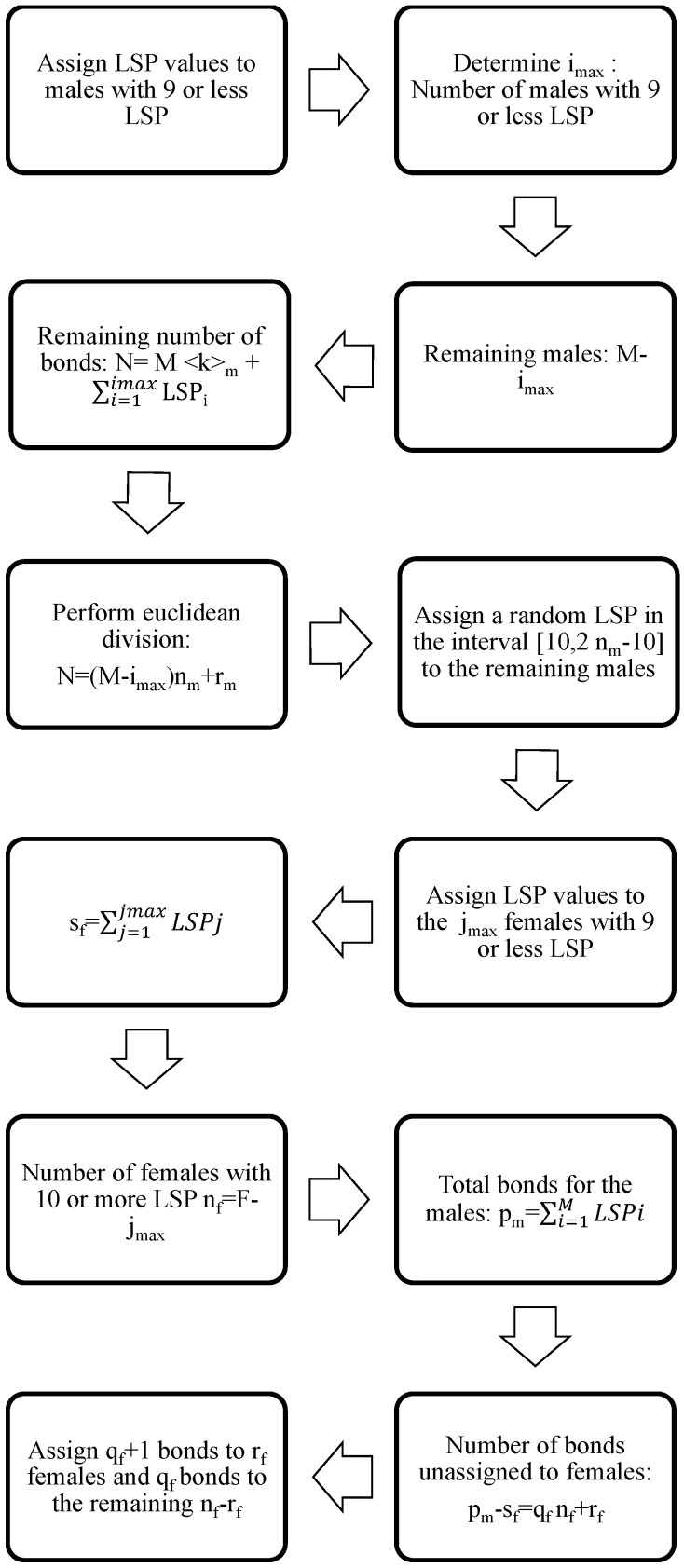
Flow diagram for the algorithm corresponding to the assignment of a number of LSPs to every male and female in the network.

Now, we denote as $p_{m}$ the total number of bonds of the male population. We must take into account, as expressed in Equation (1), that the total number of bonds of the female population should be the same. To impose that condition, we proceed as follows: (i) assign the number of bonds to the females with nine or less partners following the statistical data in Table 1; and (ii) the sum of all female LSPs in this group of $j_{\max}$ members will be denoted by $s_{f}$. Then, $n_{f}=F-j_{\max}$ is the number of females with 10 or more LSPs; and (iii) the number of bonds starting in the males and still unassigned to a female is $p_{m}-s_{f}=q_{f}n_{f}+r_{f}$, where $0\leq r_{f}<n_{f}$ and $n_{f}\geq 10$. (iv) We assign $q_{f}+1$ bonds to $r_{f}$ females still unassigned and $q_{f}$ bonds to the rest of $n_{f}-r_{f}$ females. The steps of this algorithm are also enumerated in the flow diagram in Figure 1.

Notice that, for men and women with more than 10 LSPs, we assign their LSP in the most equitable way, assuming that all of them have, more or less, the same number of LSPs. Thus, we have a lot of hubs with a low number of contacts instead of a few hubs with a lot of contacts.

From the point of view of STI transmission, the latter situation leads to a faster transmission if the hub is infected, and, also, if the hub is vaccinated, the transmission is cut faster. Therefore, due to the lack of data about people with 10 or more LSPs, we make the decision of being conservative in the transmission of the disease and in the effect of the vaccination campaigns.

Notice that this procedure implies that the condition in Equation (1) is verified. After this procedure, we have obtained the following lists:

- $AgeMale\left[i\right]$ is the age of the i-th male, i=1,…,M,
- $AgeFemale\left[i\right]$ is the age of the i-th female, i=1,…,F,
- $kMale\left[i\right]$ is the number of LSP for the i-th male, i=1,…,M,
- $kFemale\left[i\right]$ is the number of LSP for the i-th female, i=1,…,F.

These lists will be used to perform the connections of males and females and build the network. Note that, in Table 1, there are more females than males with few LSPs (comparing male and female percentages). It implies that there will be few women with a very large number of LSPs. This fact suggests us to start the assignment procedure with women with the largest LSPs. Otherwise, it would be possible that, when we have to assign LSPs of men to a female with a large number of LSPs, there will not be enough men with free sexual partners to be assigned and, for this female, it would be impossible to satisfy the condition that the degree of each node was the number of its LSP.

The assignment of partners was carried out by considering a principle of psychological similarity [24] or assortativity. Hence, we are going to define a weight function assuming that: women with few LSPs usually match men with few LSPs; people with four or more LSPs use to join with people with four or more LSPs; and couples where one of them has a large number of LSPs and the other few LSPs will be uncommon. Then, for the woman *i* and the man *j*, we define the following weight function:

(2) $$\pi \left(i,j\right)=\begin{array}{c} \left\{\begin{array}{cc} \left|kFemale[i]-kMale[j]\right| & kFemale[i],kMale[j]\leq 4\\ 0 & kFemale[i],kMale[j]>4\\ 100 & \mathrm{otherwise} \end{array}\right\},\\ +|AgeFemale[i]-AgeMale[j]-1.8|. \end{array}$$

The combined weight function, which takes into account the age difference of the partners, |AgeFemale[i]−AgeMale[j]−1.8| is defined in this way because some studies show that the average age difference among the members of a couple in Spain is 1.8 years [25].

The MSM population (around a 3.88 % of the total male population in Spain) can also be incorporated into the model, but, in this subpopulation, the connectivity would be larger than the heterosexual network. The MSM population would also be connected with the heterosexual one by links with women in such a way that every MSM individual has a link with a woman with five or more contacts [26]. The assignment of links is then performed by the a Greedy Randomized Adaptive Search Procedure (GRASP) algorithm [27,28]. Details about the construction of the whole network have been provided in previous studies [26,29].

### 2.4. The Dynamics of HPV Transmission in the Sexual Network

To implement the simulation of the transmission of HPV among the individuals in the network, a standard epidemiological model with susceptible and infected states and three types of infections (infections of high risk (HR), low risk (LR) and co-infection) was carried out. The epidemiological model is defined by the following parameters:

- We need some probabilities to determine if a sexual partner is going to produce a contagion of another partner in a given time stage. These parameters are different for each age group: 14–17, 18–29, 30–39 and 40–65. Notice that this means that the probability of contagion depends upon the age group of the members of the relationship. Moreover, the probability of connection of these members in the network is also age-dependent as proposed in Equation (2). The values of these probabilities are determined in the process of the model fitting.
- Average time an individual infected by a HR HPV clears the infection and recovers.
- A similar parameter for clearing the LR HPV infection.
- If a partner produces the contagion of his/her partner, we need another four parameters to determine if the high or low risk HPV infection is transmitted from man to woman and vice versa.

Another additional parameter is necessary to generate the network. This is the average number of LSPs for men (parameter *k*). Simulations are run by generating a network and carrying out a large number of epidemic evolution time-steps starting with a number of individuals infected by both HPV types as given by the CLEOPATRE study [3]. After the warm-up period, we obtain a stable situation and we can proceed with the calibration by comparing the model predictions with real data and deducing the most probable values of the set of parameters.

We have used a calibration procedure using the Particle Swarm Optimization (PSO) algorithm. The prevalence data for each age group is listed in Table 2:

Note that the network building and the transmission parameters involve randomness and uncertainty due to the random processes used in the network building and the transmission dynamics of the HPV. This fact is going to be taken into account in the calibration and simulation.

To check the reliability of the model and the calibration, we simulated the HPV vaccination campaign carried out in Australia [8], and compared them with the actual impact published [8]. In 2007, Australian health authorities started a vaccination program for 12–13 year-old girls with a coverage of 73% (83% in the first dose, 80% in the second dose and 73% in the third dose). In addition, from 2007 to 2009, there was a catch-up vaccination program for women aged 13–26 with a decreasing coverage with age until 52% in women aged 20–26. Their results can be summarized:

- Two years after the vaccine was introduced, the proportion of GW diagnosed declined by a 59% in vaccine eligible young women aged 12–26 years in 2007, and by 39% in heterosexual men of the same age.
- No significant decline was observed in women or men older than 26 years old, non-resident young women, or men who have sex with men.

Two different scenarios were considered to be simulated:

- Scenario 1: vaccination of 83% of the 14 year-old girls (or younger girls) plus a catch-up with coverage 73% for 14–26 year-old women.
- Scenario 2: vaccination of 73% of 14 year-old girls (or younger girls) plus a catch-up with a vaccination coverage of 52% for 14–26 year-old women.

These simulations represented the upper and lower bounds of the scenario implemented in Australia. The assumed effectiveness of the vaccine was 96.5%.

Due to the randomness of the simulations, we will show the average and confidence intervals among the 30 simulations per every scenario that we have considered above. This number was chosen as a compromise among efficiency of the method and computational feasibility. We have found that, with these runs, we obtain reasonable parameters in many of the simulations. Of course, it would be useful to increase this number, but this cannot be achieved, in a sensible computing time, with the computational resources that we devoted to the task. For example, a single run takes 162 h per run for a network of 500,000 nodes and 256 h in the case of 750,000 nodes (in a single processor of a Sandy Bridge platform). Simulations were run on a multi core platform with 64 processors and 500 GB RAM and every processor was assigned with the computation of a simulation for a given set of parameters.

### 2.5. Calculation of the Number of Infections

We call *I* the number of infected women of LR HPV 6 and/or 11 just before the starting of the vaccination campaign; we call $V=(v_{1},\ldots ,v_{N})$ to the number of infected women of LR HPV 6 and/or 11 every month from the starting of the vaccination program until the end of the simulation. Then, the vector

(3) $$100\times \left(1-\frac{v_{1}}{I},\ldots ,1-\frac{v_{N}}{I}\right)\;$$

is a measure of the percentage of decline of number of infected women of LR HPV 6 and/or 11 after the beginning of the vaccination campaign. This will also be applied to men and MSM.

In order to compare GW data given in [8] with our model, results referred to infected women of LR HPV 6 and/or 11, we should take into account that, whether a fixed proportion of HPV 6 and/or 11 infected individuals develops warts, the percentage of decline in warts and in infected women of LR HPV 6 and/or 11 will be comparable.

Another important issue for the natural history of the disease is the persistence of the infection [30]. Our model does not consider the persistence “a priori”, but we derive the cases of genital warts from the number of cases of infected individuals by taking this data into account.

## 3. Results

After calibration, the model predicted an average number of LSPs in men of 7.7; confidence interval 95% (CI95%) (7.3,8.5), with an average duration of an infection due to LR genotypes of 0.5 CI 95%(0.3,0.8) years, for men and women.

The transmission probability from women for LR genotypes is 0.58 CI 95%(0.57,0.59). From men, it is 0.63 CI 95%(0.49,0.72).

### The Australian Scenario

**Figure 2:**
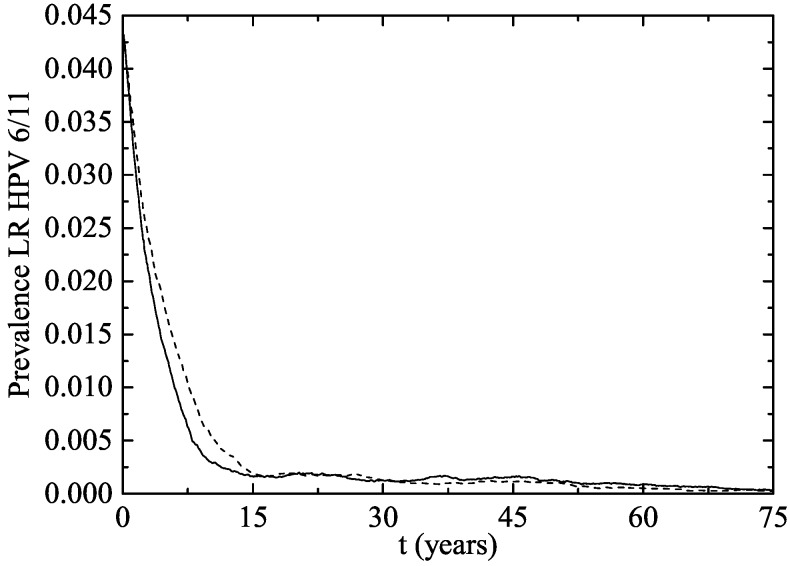
Percentage of women aged 14–26 infected of LR HPV 6 and/or 11 after the implementation of the vaccination program (Scenario 1: solid line and Scenario 2: dotted line).

Figure 2 shows the percentage of women aged 14–26 infected after starting the vaccination program in both simulated scenarios. The fast decrease in both scenarios can be seen at the very beginning.

**Figure 3:**
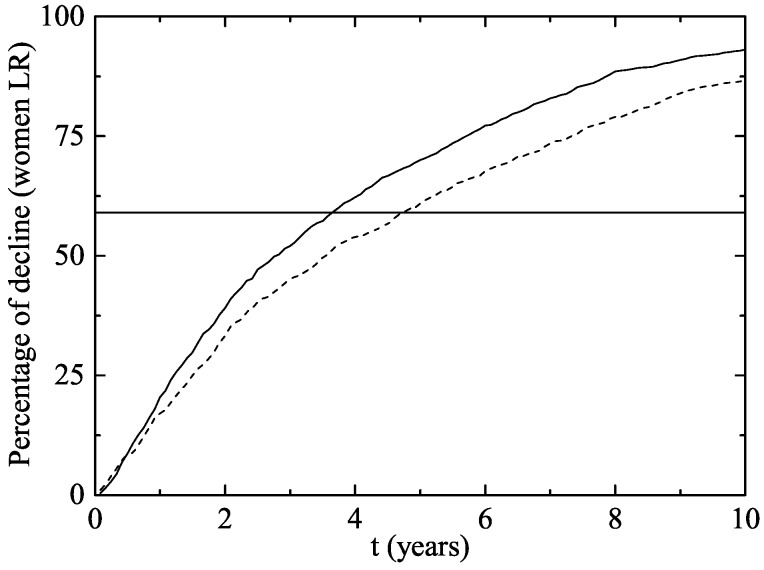
Percentage of decline of women aged 14–26 infected of LR HPV 6 and/or 11 (and consequently of GW) after the implementation of the vaccination program in both scenarios (Scenario 1: solid line and Scenario 2: dotted line). The horizontal line represents the percentage of decline in Australian women wart cases after two years.

In Figure 3, we have plotted the same data as in Figure 2 but from another point of view: the average percentage of decline of women infected of LR HPV 6 and/or 11. As the vaccination program progresses over time, the percentage of decline obviously grows.

In our model simulation, after two years of the beginning of the vaccination program, a decline of 33.3–39.1% has occurred in women and 3.6–4.6 years were necessary to reach the Australian 59% decline rate in GW.

**Figure 4:**
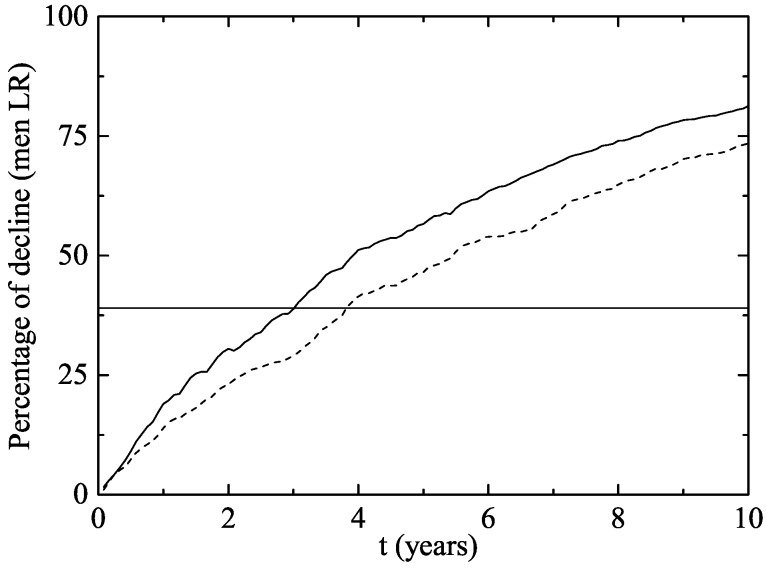
The same as Figure 3 but for men aged 14–26.

**Figure 5:**
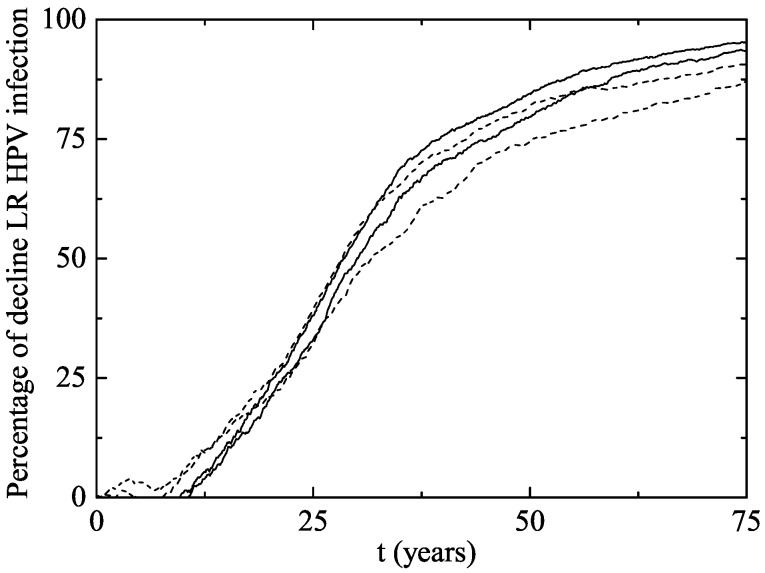
Percentage of decline of men and women aged 27–64 infected of LR HPV 6 and/or 11 (and consequently of GW) after the implementation of the vaccination program in both scenarios. The upper solid line corresponds to women in Scenario 1 and the lower solid line corresponds to women in Scenario 2. The upper and lower dotted lines correspond to men in Scenarios 1 and 2, respectively. Notice that no significant decline is observed in women or men aged 27–64 in almost the first 10 years.

In men aged 14–26 (Figure 4), there was a decline of 23.1–30.5% after two years and 3–3.75 years were necessary to reach the Australian 39% decline rate of infection. No significant impact on the rate of infection was observed in women or men aged 27–64 in the first 10 years after the implementation of the vaccination program (Figure 5). It can be explained by the fact that, usually, individuals have sexual intercourses with people more or less the same age.

**Figure 6:**
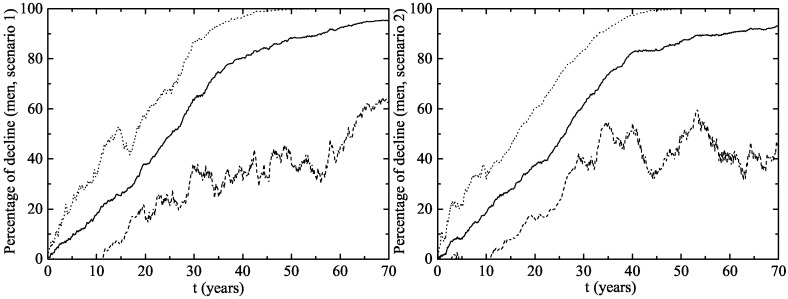
Herd immunity effect of the vaccination program in Australia on heterosexual men (**left** figure (Scenario 1) and **right** figure (Scenario 2)). The upper dotted and the lower dashed lines correspond to an interval of 90% confidence. The solid line is the average evolution of the decline in GW.

**Figure 7:**
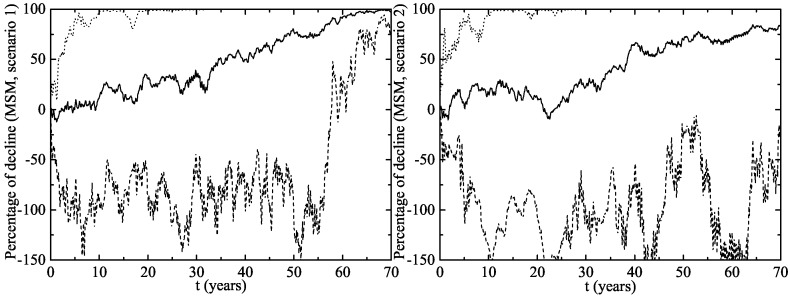
The same as Figure 6 but for MSM.

**Figure 8:**
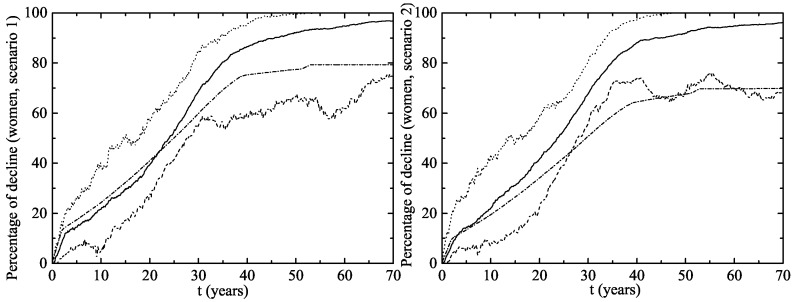
Percentage of decline in GW cases for women for the vaccination program in Australia (**left** figure (Scenario 1) and **right** figure (Scenario 2)). The upper dotted and the lower dashed lines correspond to an interval of 90% confidence. The solid line is the average evolution of the decline in GW for the whole female population and the dashed-dotted line is percentage of vaccinated women. Notice the herd immunity effect also contributes to the decline in the number of infections for unvaccinated women. This can be seen when the decline lines are over the vaccination line.

The herd immunity effect in both scenarios is shown in Figure 6 for heterosexual men and Figure 7 for MSM. Notice that, in men and MSM, any decline is due to herd immunity. The decline of GW in the whole female population is given in Figure 8. This is predicted when the lines representing their decline are over the vaccination line also shown in this figure. We see that the herd immunity effect starts after 2.58–2.91 years when 11.2–14.45% of women are vaccinated.

Notice that the herd immunity effect is very clear within the 90% CI both for heterosexual men and women, but it is uncertain in the MSM population. In the best case scenario, the MSM subpopulation achieves a large protection level, but there are other situations in which it remains largely unprotected and the HPV strains still circulate among them for many years. This could be attributed to the way in which the MSM individuals are connected: with a very large number of LSPs among them and some casual links with women with large LSPs. If these women, acting like hubs in the network, are vaccinated, we obtain a fast eradication of the disease in the MSM population and this would be the best scenario.

## 4. Discussion

The random network of sexually transmitted HPV including up to 500,000 nodes was developed to fit the data of surveys concerning the number of sexual partners throughout life [26,29]. Standard continuous models are insufficient to accurately predict transmission because they do not account for the individual to individual transmission of the infection, the role of hubs in disseminating the virus through the rest of the population and nor the vaccination campaigns targeting specific groups of individuals.

This network has successfully been applied to the stable state of infections by LR and HR HPV genotypes in Spain [26]. In this study, we mimicked the results found in the HPV vaccination campaign in Australia [7,8] and showed very reliable results.

Model predictions in key epidemiological data was always within the range of the published results. Thus, the 7.7 number of LSP is close to the 8 published in Spain [31]. In addition, the average duration of infection for LR genotypes is similar to the reported, which is 0.5–1.12 for the LR genotypes [5,12,32]. The transmission probability from women and men is also closed to the parameters used previously [5,32]. All of this reassures the reliability of the model.

Models based upon continuous differential equations predict a slower decrease in the number of infected individuals after implementing similar vaccination campaigns [5,6]. Hence, the case of the HPV vaccination in Australia provides one of the best real scenarios for testing new network models in mathematical epidemiology. There is an on-going debate on the pertinence of an approach based upon networks on epidemiology [33], and this work contributes to show the necessity of such an approach in many cases, in particular, in those corresponding to STI.

To validate the model, we used the Australian experience, with two different vaccination coverages: routinely vaccination campaign for 12–13 year-old girls with a coverage of 73% and 83% and a catch-up program in the 14–26 age group with an average coverage of 52% and 73%. This program revealed an important herd effect [8], so that vaccination decreased the incidence of GW even in the non-vaccinated men because of the protection of infection conferred by the vaccine, and the decreased transmission of the virus.

The model predicted a fast decline in the number of infections parallel to the decline in the number of GW in Australia. However, this model was built with Spanish data on sexual behavior [18] and prevalence of HPV infection [3], which might differ to the Australian one, and may explain the minor differences found between the model and the actual data published. Another potential cause of these differences could be the need for a three dose schedule that we simulated, when it has been proposed that with one dose the short term protection against GW is practically 100% [8]. Herd immunity in this model of STI is predicted much sooner than in other highly transmitted aerial transported infectious diseases as influenza or respiratory syncytial virus, due to the structure of the network. This supports the need to build appropriate LSP networks.

Other models have also predicted the protection of males by vaccinating girls and women, but only for heterosexual men, as the model used by Bogaards et al. [9]. This model uses Bayesian techniques to study the herd immunity effect. However, in contrast with our model, it does not take into account the dynamics of the HPV transmission, the importance of age-groups and the different roles they play in the propagation of these viruses or the links among the MSM subpopulation and the heterosexual network. In this sense, a network model is required to study the impact of the vaccination strategies in short, medium and long time scales.

Vaccination strategies should seek an optimal effectiveness and efficiency. The impact of vaccination in males should always consider the herd immunity of vaccinating girls. However, it is shown that vaccination of women leaves heterosexual males only partially protected at least in the first 10 years after vaccination. However, MSM take 50 years on average, in order to have a decrease in the incidence of infection of 50%. This can be the consequence of the large LSP numbers for MSM and their casual connections with women with large LSP numbers in the heterosexual subnetwork.

The model considers a quiet close community, where there is not much contact with other communities. This may not be the case in Spain, which in 2016 received over 75 million tourists [34], representing almost double the number of Spanish inhabitants, and when sexual contact is frequent. This may bias the results, as the herd immunity in Spain may not be so clear as in countries with less tourism.

Another issue that we must take into account is the modelling of the population with a high number of contacts because these individuals are hubs in the network whose vaccination may induce a faster decline of the virus prevalence. Our approach is rather conservative in the assignment of LSP for men and women with 10 or more links because we assume that all of them have similar LSP. However, it is expected that individuals with extreme values of LSP are favouring the transmission of HPV in such a way that a targeted vaccination can show its benefit in a very short time.

## Figures and Tables

**Table 1:** Proportion of males and females per number of life sexual partners LSP per age group.

**MALES**						
**Age**	**0 LSP**	**1 LSP**	**2 LSP**	**3–4 LSP**	**5–9 LSP**	**10 or More LSP**
14–29	0.107	0.207	0.131	0.225	0.168	0.162
30–39	0.027	0.225	0.128	0.21	0.17	0.24
40–65	0.019	0.268	0.14	0.193	0.163	0.217
**FEMALES**						
**Age**	**0 LSP**	**1 LSP**	**2 LSP**	**3–4 LSP**	**5–9 LSP**	**10 or More LSP**
14–29	0.138	0.43	0.186	0.158	0.056	0.032
30–39	0.029	0.501	0.168	0.177	0.077	0.048
40–65	0.017	0.652	0.138	0.118	0.039	0.036

**Table 2:** Prevalence of HR- and LR-infected women per age groups from the CLEOPATRE study [3]. Co-infections are included in both HR- and LR-infected, mean and 95% confidence intervals.

Women	HR-Infected	LR-Infected
18–29 y.o.	24.10%, [21.33%,26.98%]	6.36%, [4.71%,8.07%]
30–39 y.o.	11.01%, [7.54%,15.09%]	1.26%, [0.0%,3.14%]
40–64 y.o.	5.96%, [4.29%,7.8%]	2.37%, [1.22%,3.68%]
18–64 y.o.	16.23%, [14.52%,17.97%]	4.41%, [3.42%,5.45%]

## Supplementary Materials

Further details and results about the building of lifetime sexual partner networks can be found here: lsp.imm.upv.es.

## Abbreviations

The following abbreviations are used in this paper:

VLP	Virus-like particles
STI	Sexually transmitted infection
HPV	Human papillomavirus
LSP	Lifetime sexual partner
HIV	Human immunodeficiency virus
PSO	Particle Swarm Optimization
MSM	Men having sex with men
GW	Genital warts
